# The Position and Prospects of Hospitals in 1912

**Published:** 1911-12-30

**Authors:** 


### THE POSITION AND PROSPECTS OF HOSPITALS IN 1912.

### Mr. Walter Alvey Interviewed on the Outlook.

The New Year is a turning point in hospital history in view of the situation created by the passing of the Insurance Act, which is expected by those responsible for it to come into force at the early date of July 15, 1912, or some subsequent date not later than January 1, 1913. At this critical moment Mr. Walter Alvey has consented very courteously to illustrate the position in which London hospitals find themselves by summing up the efforts made by the hospital of which he is secretary during the past year, and by giving his opinion on the general outlook. This he has given special point to by reference to the difficulties that have been created in the way of the appeal which was issued under Lady Juliet Duff's signature on behalf of Charing Cross Hospital some months ago.

"It was unfortunate, of course," said Mr. Alvey, "that the issue of our appeal coincided with that of the Middlesex Hospital, but that is one of those difficulties with which any hospital, and any secretary, may be confronted. It simply could not be helped, so we went ahead and asked boldly for £100,000. During the year we have received, it is true, only £5,500, but I would ask you to consider the way in which that money has been raised.

### Contrasted Methods of Appeal.

"We have confined ourselves almost entirely, up to the present, to personal appeals in one form or another. Of course, I am well aware that there are great possibilities in the organising of big entertainments and social functions, but in view of what was being done by Middlesex Hospital in that direction, and considering also the number of social events with which this particular year was likely to be crowded, we thought it advisable not to attempt anything of that nature ourselves. Taking all the circumstances into account, I don't think the result is bad, particularly when you look at what it has enabled us to do."

"Ah, yes; how has the £5,500 been employed?"

"It has been added to the money we had already accumulated in the sinking fund, and by this means we have paid off £20,000 of our mortgage debt, and have effected a saving on our payments for interest and sinking fund of £620 per annum. So you see, though we have not accomplished as much as we could have wished, the year has not really been a barren one."

"How about the Coronation?"

"Oh, the Coronation," said Mr. Alvey, laughing, "that of course upset everybody, and though more than one person said that Coronation year ought to be a good year for charity, I cannot say that I think events have proved them to have been right."

### The Effect of the Insurance Act.

When asked as to the effects produced already by this measure, Mr. Alvey said, "The Bill, now, I regret to say, an Act, has not bothered us a great deal so far. We have, it is true, received one or two refusals, on the ground of this particular legislation, from people who were asked to become new subscribers, and I have heard rumours of withdrawals next year on the part of old supporters, but it is too early yet to speak with any certainty as to the effects of the Act. My own impression is that the great mass of people will not realise what the Act means to them until it actually comes into operation, and that hospitals, therefore, will not feel its effects very severely until that time arrives."

"What will insurance cost Charing Cross?"

"We shall have to pay about £70 a year for the insurance of our employees. But that is on the supposition that all the employees consent to pay their own contributions. If the Servants' Anti-Tax movement spreads to hospitals we may have to pay a good deal more."

"Are you in favour of a State contribution to hospitals?"

"I am in favour of a *pro rata* payment for every insured case that we treat. The only thing the Government offers us is an uncertain contribution which we may or may not be able to obtain from the Health Committees to be appointed under the Act. This is not in the least what we want."

### "Refuse to Treat Insured Cases!"

"The hospitals should adopt this as a policy?"

"Yes; I am definitely in favour of the voluntary hospitals combining together and saying as a body 'We will not receive nor treat any insured case till proper provision has been made by the Government for the payment necessary for this work.' This may seem a very harsh policy to adopt, but I believe it would be the quickest way of bringing about a proper solution of the question, because it would speedily prove to the nation at large what a fatal mistake the Chancellor of the Exchequer has made in leaving the voluntary hospitals out of the Act. Quite apart from that, however, I question whether hospitals will be justified in using the money given them by the charitable in treating those whom the State has undertaken to provide for. It seems to me that it would be a distinct misapplication of funds.

"Personally, and I know there are others who hold the same view, I should like to see the whole question of the future policy of the hospitals in relation to this Act dealt with by the three hospital funds. Any course of action that they might decide upon would, I feel sure, commend itself to most London hospitals, and the general mass of subscribers, too, would have confidence in their judgment. It is desirable, however, to bring all hospitals throughout the kingdom into line, and I suggest this might be done if the Funds would combine with the British Hospitals Association in this matter. That Association is certainly the only body that has made any sustained effort to obtain for us recognition in the Bill."

"Do you agree with the proposed campaign among working men?"

"I am not at all sure that this is work which the hospitals should engage in. There is a great difference between deciding upon the line of policy they themselves should adopt, and entering into an active political campaign. Still, this is a question which affects chiefly the provincial hospitals, as they depend to a much greater extent than we in London upon the working men's support."

"Will that support be permanently affected?"

"Opinions differ, I know, as to that," Mr. Alvey retorted. "Personally, I think it will, and that all our sources of income will suffer far more than a passing reaction. And that reminds me that another difficulty which the Charing Cross Hospital has had to contend with this year has been the scarcity of legacies. We cannot, of course, attribute this to the Insurance Act, but it must be remembered that the kind of legislation of which this Act is a recent example dates back for some years."

The General Outlook.

"You see our general position. What is true of Charing Cross is true in varying degrees of all the hospitals, and if we are to win through it is absolutely imperative that we should have a united policy."

"If your appeal is successful next year?"

"In that fortunate, but improbable event, our five empty wards would be at once opened—and at once filled."

"You would never entertain the question of moving?"

"No; for the simple reason that though you can move the hospital you can never move Charing Cross. If we lost our name we should lose everything. Besides, there is no doubt that the hospital is wanted where it is."

"Is there any chance of an amending Act being passed?"

"That is easily answered," replied Mr. Alvey, laughing, "what time has the Government for it? The Act comes into force next July, or January 1913 at latest, and in the interval the Government proposes to deal with Home Rule, Disestablishment, and Manhood Suffrage. That is enough, I should have thought, to keep any Government's hands full. The outlook for our appeal in 1912, and for the successful carrying on of the voluntary hospital system generally, is not, I must admit, very encouraging, but I think the future is largely in our own hands, and that much depends upon what the hospitals decide to do now."

